# Problematic Use of Pornography: Clinical Presentations, Diagnosis and Interventions

**DOI:** 10.1192/j.eurpsy.2023.144

**Published:** 2023-07-19

**Authors:** S. Virani

**Affiliations:** Psychiatry, University of Massachusetts Chan Medical School, Worcester, United States

## Abstract

**Abstract:**

The COVID-19 pandemic has caused immense psychosocial strain worldwide. Excessive use of the internet during these psychologically trying times, fueled by physical isolation as a result of lockdowns, translated into dysfunctional behaviors. A growing body of evidence suggests an unprecedented increase in internet use and consumption of online pornography during the pandemic, and possibly even directly caused by it. This presentation will focus on the the statistics, variations in diagnostic criteria, clinical presentations and interventions for problematic online pornography use. Practical solutions will be offered to show how foresightedness with utilizing existing tools and therapies and exercising appropriate amounts of caution could go a long way in addressing the challenges that lie ahead in the post-pandemic era.

**Disclosure of Interest:**

None Declared

